# Anatomy education at central Europe medical schools: a qualitative analysis of educators’ pedagogical knowledge, methods, practices, and challenges

**DOI:** 10.1186/s12909-025-07722-6

**Published:** 2025-08-18

**Authors:** Jennifer Valcke, Laura Berta Csík, Zoe Säflund, Andras Nagy, Amani Eltayb

**Affiliations:** 1 Department of Learning, Informatics, Management and Ethics, Karolinska Institutet, Solna, Stockholm, Sweden; 2 Student Services, Medical School, University of Pécs, Pécs, Hungary; 3 Department of Anatomy, Medical School, University of Pécs, Pécs, Hungary

**Keywords:** Anatomy teaching, Student-centred pedagogy, Constructive alignment, ILOs, SDG4.7, Qualitative

## Abstract

**Abstract:**

Globally, there has been a growing demand for a unified education standard, spurred by sustainability initiatives such as the United Nations’ Agenda 2030 and the increasing internationalisation of higher education. The World Federation for Medical Education (WFME) promote accreditation process for international medical education institutions that provide curricula in English. However, some Central European Medical Schools offering such curricula are not fully aligned with WFME accreditation standards. Organisers of human anatomy courses at these schools are seeking to improve their skills and abilities to deliver high-quality teaching effectively in multicultural and multilingual environments. A survey conducted by the Erasmus + Strategic Partnership project LEANbody, which aims to reach for quality management tools to teach human anatomy effectively in a multicultural and multilingual learning space, revealed that over 70% (49/69) of anatomists in Hungary, the Czech Republic, and Croatia are unfamiliar with international quality standards for medical education and the concept of student-centred pedagogy.

**Aims:**

This study seeks to understand educators’ perceptions of pedagogical knowledge and concepts/frameworks, such as constructive alignment (CA), Intended Learning Outcomes (ILOs), in relation to student-centred pedagogy and their anatomy teaching practices. The study also investigates perceived gaps at the institutional, departmental, and individual levels concerning anatomy teaching and the pedagogical practices that should be promoted.

**Methods:**

A descriptive cross-sectional study using a qualitative approach was used for this purpose. In 2022, face-to-face or online interviews were conducted with 14 anatomy educators including course organisers from Zagreb, Masaryk and Pécs Universities.

**Results:**

We found that most educators had not received formal teacher training on teaching methods prior to starting anatomy teaching and were unfamiliar with such pedagogical frameworks as CA, even though they were familiar with the concept of ILOs. Thematic analysis was applied to open-ended questions and the umbrella theme that emerged was “Transforming Anatomy Teaching and Learning in the Glocal Classroom: Navigating the Intersections of Pedagogical Practice, Constructive Alignment, and Student-Centred frameworks”. Two themes and 5 subthemes were identified from the data. The study presents recommendations and a novel framework linking student-centred approaches, CA, and global educational sustainability agendas, such as the sustainable development goal 4 target 7 (SDG4.7) to enhance the quality of anatomy teaching.

**Supplementary Information:**

The online version contains supplementary material available at 10.1186/s12909-025-07722-6.

## Background

The internationalisation of higher education has undergone significant changes over the past two decades. Initially, it involved ad hoc activities and initiatives, but it has now shifted toward integrating international dimensions into the curricula of Higher Education Institutions (HEIs). This broader approach aims to enhance and improve programme delivery [1]. Notably, the internationalisation of education equips graduates with competences to address societal challenges [2], moving beyond mobility and teaching in English [3]. Sustainable Development Goal 4, target 7 (SDG4.7) [4] highlights that all persons involved in education, including teaching staff, should align education with global citizenship, human rights, gender equality, cultural diversity, and the role of culture in sustainable development [5]. In addition, the Organisation for Economic Co-operation and Development (OECD) encourages HEIs, including medical schools, to equip graduates with the necessary knowledge, skills and attitudes for a globalised world [6, 7]. In alignment with sustainability agendas and the global efforts for the internationalisation of higher education, the World Federation for Medical Education (WFME) aims to ensure that accreditation of medical education institutions offering curricula in English meets high international standards [8]. Learning objectives for anatomy, when outlined through the WMFE (Well-Defined, Measurable, Focused, Explicit) framework, are constructed to ensure clarity, accessibility, and direct alignment with instructional and assessment strategies. Medical schools become accreditation compliant by meeting the standards set by their respective accrediting agencies. These agencies must have WFME Recognition Status for their accreditation to be internationally recognized. Currently, Central European medical schools in this category are not yet eligible for WFME accreditation. Human anatomy course educators at these schools need to develop skills that will allow them to harness the potential of multicultural and multilingual learning environments to improve the quality of teaching and learning. Additionally, these educators must also align their efforts with key international frameworks, such as Objective 1.1 of the WHO’s *Global Strategy on Human Resources for Health: Workforce 2030* [9], the Professional Standards Framework [10], and SDG4: Quality education [4].

A needs analysis was prepared through the Erasmus + Strategic Partnership project called LEANbody “LEAN in Medical Education: Reaching for Quality Management Tools to Teach Human Anatomy Effectively in a Multicultural and Multilingual Learning Space: Project number: 2021-1-HU01-KA220-HED-000027542”. A need analysis survey conducted among anatomy educators in Hungary, the Czech Republic, and Croatia revealed that over 70% (49/69) of educators were unfamiliar with international quality standards in medical education [11]. Most had never encountered concepts such as student-centred pedagogy or assessment methods designed to measure the professional development of their students or themselves. Additionally, despite many being medical doctors, they admitted to having limited knowledge of workplace mental health management principles. This project recognises the linkage between SDG4.7 [4] and the internationalisation of higher education [2] as both emphasise quality education, making a meaningful contribution to society through global citizenship. To achieve these goals, the LEANbody project advocates for student-centred pedagogy, action-oriented learning, and transformative teaching.

Considering the above context, the critical questions for LEANbody is: Are anatomy educators adequately prepared to provide high quality teaching and learning that align with global educational standards and sustainability agendas? Are these educators formally trained to equip graduates with the necessary knowledge, skills, and attitudes for a globalised world? Are they aware of pedagogical approaches and principles such as student-centred learning, constructive alignment (CA), and ILOs among others? Do they apply these pedagogical principles to their practices? What are the potential gaps as perceived by educators at the institutional, departmental, and individual levels with regards to anatomy teaching?

This article is based on the results from the needs analysis and answers the above questions and describes how the LEANbody project explores anatomy teaching and learning in a multicultural and multilingual context and provides recommendations on how to enhance the quality of anatomy teaching in Central European medical schools, specifically at the University of Pécs in Hungary, Masaryk University in the Czech Republic, and the University of Zagreb in Croatia.

### Pedagogical concepts and frameworks

**Fig. 1:**
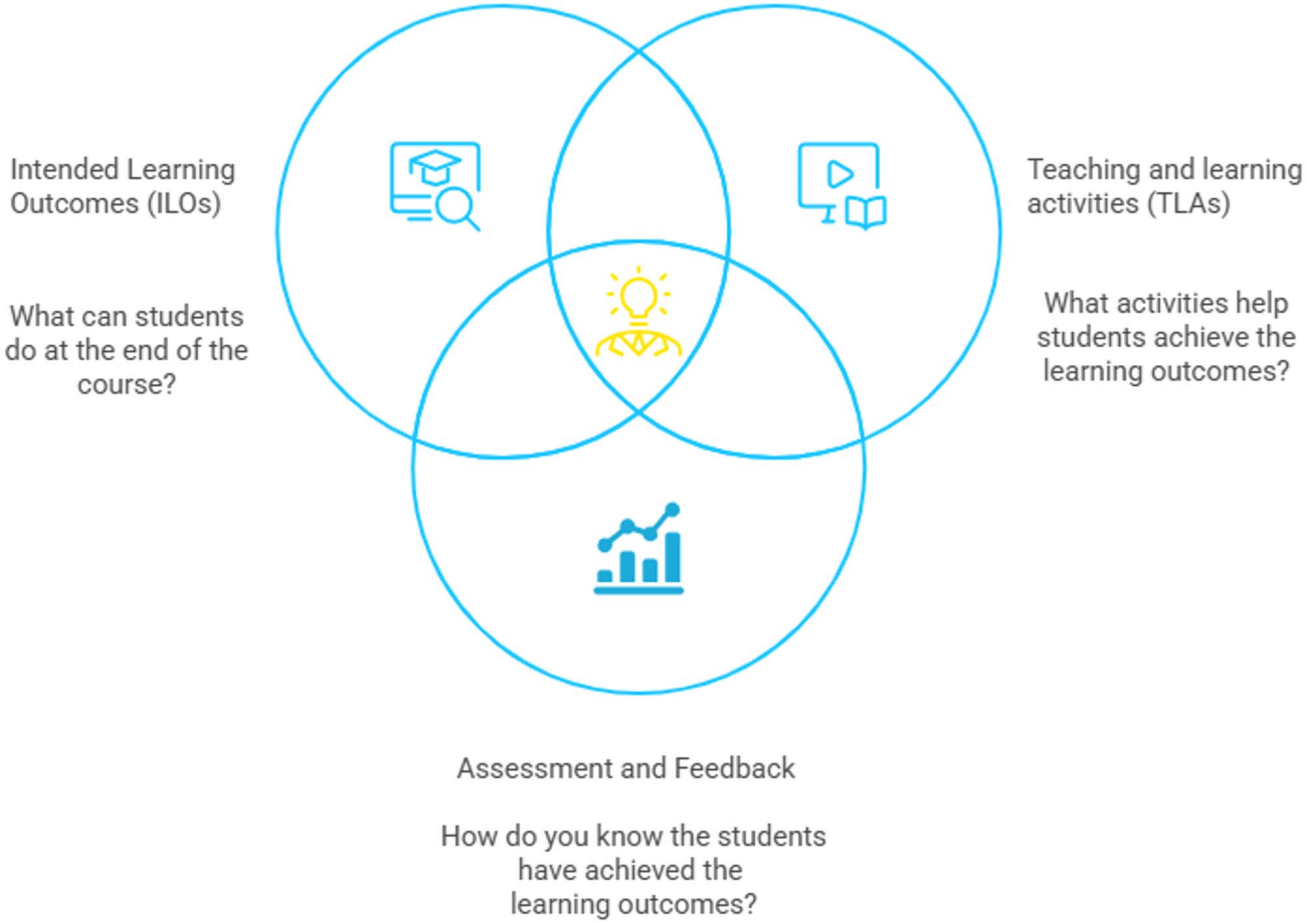
The Principles of Constructive Alignment (CA)-Figure created in Napkin

In today’s multicultural and multilingual classrooms students should adopt a “glocal” (local and global) approach, thinking globally while acting locally. Educators are expected to develop skills and competencies essential for navigating teaching and learning in these “glocal classrooms”. Glocal competencies are defined as the ability of educators to engage with students in ways that harness the potential benefits of linguistic, cultural, social, and other forms of diversity among students and between students and educators [12, 13]. This requires aligning teaching and learning methods and practices to global standards as internationalisation and SDG4.7. The internationalisation of higher education is defined as the “intentional process of integrating an international, intercultural, and/or global dimension into the purpose, functions, and delivery of post-secondary education. This aims to enhance the quality of education and research for all students and staff, and to make a meaningful contribution to society” [2, 14]. This definition was originally developed by Professor of Education Jane Knight in 1993 and has been refined over time. It was not provided by a specific governing or accrediting body. Rather, it has been widely adopted and cited in academic literature and by various international education organisations. This definition connects with the SDGs, particularly SDG4.7, which defines quality education as inclusive and equitable. It emphasises a lifelong learning perspective that considers all stakeholders, including teaching staff, as learners [4]. The interconnections between the internationalisation of higher education and SDG4.7 is clear and provide us with useful frameworks that define quality education. They involve integrating international, cultural and global dimension into the curriculum, while also incorporating the principles behind SDG4.7, among which human rights, gender equality, a culture of peace and non-violence, global citizenship, and an appreciation of cultural diversity. Such integration requires careful alignment which can be applied through the CA framework, an outcome-based framework to teaching that closely aligns ILOs, Teaching and Learning Activities (TLAs), as well as assessment and feedback practices. ILOs are statements written from the students’ perspective, indicating the level of understanding and performance they are expected to achieve through engagement with the TLAs [15]. ILOs define the TLAs students need to engage in, while assessment and feedback practices evaluate how effectively students perform these activities [16]. The three pillars of CA should be clearly communicated to students before teaching begins. CA plays a crucial role in the teacher-student relationship by clarifying both what students are expected to learn and how they will achieve it. Additionally, CA supports learning by guiding students in acquiring competencies aligned with their learning goals. By providing clear and objective feedback on performance, CA gives students confidence in their ability to assess their own progress accurately, as shown in Fig. 1 below. In Europe, anatomy teaching has undergone significant developments, with a mix of traditional and modern approaches being employed to enhance learning outcomes. Human anatomical donor dissections and didactic lectures remain a cornerstone of anatomy education. These methods are valued for providing hands-on experience and a comprehensive understanding of the human body in situ. The integration of digital tools, 3D modelling, virtual simulations, and interactive multimedia has become increasingly prevalent. Many institutions now combine traditional dissection with modern digital tools to provide a more versatile learning experience that caters to diverse student needs. While there is no unified formal teacher training program for anatomy educators across Europe, advancements in teaching methodologies highlight the need for such specialized training to ensure effective delivery of both traditional and modern educational practices. Formal teacher training specifically tailored to anatomy teaching is not widely standardised across Europe. Some courses exist to train educators in general medical teaching methodologies, such as the “Teach the Teacher” course, which provides continuing professional development (CPD) accreditation for medical professionals interested in teaching. Several countries and medical schools have adopted CA in their curricula with different training approaches. For instance, King Saud bin Abdulaziz University for Health Sciences and the United Arab Emirates University use a 12-step guide for curriculum alignment. Karolinska Institutet has incorporated CA into their Internationalisation of the Curriculum (IoC) project. Even though there is no standardised formal teacher training program for anatomy educators across Europe, evolving teaching methodologies underscore the growing need for specialised training to effectively integrate both traditional and modern educational practices. Giving the evolving landscape of anatomy education, with a growing emphasis on clinical application, communication and humanistic skills, teacher training has become even more crucial. It prepares educators to effectively teach in ways that foster these competencies, aligning with the broader goals of healthcare education. Therefore, this study aims to explore educators’ pedagogical knowledge and practices in teaching and learning anatomy in relation to international quality standards for medical education and the concept of student-centred pedagogy. Additionally, we investigate educators’ perceptions of gaps within their institutions.

### Reflexivity statement

The authors of this article hail from Karolinska Institutet and Pécs University, bringing diverse expertise in medical education, with a particular focus on anatomy education. Coming from different institutions and disciplinary cultures can impact the data both positively and negatively. On the positive side, it brings diverse perspectives and allows us to interpret the data through various lenses. However, it might also introduce biases, as we may view the data through the lens of our local context and institutional affiliations, potentially affecting our interpretation. In this endeavour, we leveraged our collective knowledge and expertise in medical and educational development, working collaboratively to ensure our study was valid, sensitive, and appropriate within the context of anatomy education.

## Methods

In this study, researchers of the Erasmus + Strategic Partnership project LEANbody collected both demographic and interview data from anatomy educators to answer the research questions.

### Study design

A descriptive cross-sectional study was conducted using a qualitative approach. Data were collected through semi-structured interviews between April and December 2022.

### Study participants

Participants were anatomy educators with teaching responsibilities at the anatomy departments of the University of Pécs in Hungary, Masaryk University in the Czech Republic, and the University of Zagreb in Croatia during the period of this study. These educators are also involved in the LEANbody project. Data collected is accurate and representative as we included anatomy educators from different universities in central Europe.

Out of the 15 anatomy educators who were approached to participate in the study, 93% responded (14/15). Most of these educators primarily taught in their local language alongside English. Among the 14 educators, 9 (64%) were male, and 5 (36%) were female. On average, 57% (8/14) of educators are experienced teachers with more than 10 years of teaching experience, while only 14% (2/14) were new or relatively new to teaching (up to 5 years of experience), as shown in Table 1 below.

**Table 1:** Socio-demographic characteristics of educators and their pedagogical practices

Questions	Total *N* of educators 14			
	Gender *N* (%)			
	Male 9 (64%)		Female 5 (36%)	
	Years of teaching experience N(%)			Total N (%)
	Novice0-5 Years, 2 (14%)	Medium experience 6-10 Years, 4 (29%)	Expert >10 Years, 8 (57%)	
Experience of formal pedagogical training (YES/NO)	NO 2 (100%)	NO 4 (100%)	NO6 (75%)	NO 12 (86%)
Knowledge about constructive alignment (YES/NO)	NO 2 (100%)	NO 3 (75%)	NO5 (63%)	NO 10 (71%)
Knowledge about ILOs (YES/NO)	YES 1 (50%)	YES 2 (50%)	YES6 (75%)	YES 9 (64%)
Experience of drafting ILOs (YES/NO)	NO 2 (100%)	NO 4 (100%)	NO4 (50%)	NO 10 (71%)

### Data collection

In total, 15 anatomy educators were approached using purposeful sampling [17]. While all 15 educators had agreed to participate in the study, one educator was unavailable during the data collection period. A researcher and a trained administrator in interview techniques collected data using interviews, both face-to-face or online. The data was collected following an interview guide developed by a trained psychometrician in discussion with the researcher and the administrator. Each educator was informed about the study’s aims and provided written consent. The written informed consent signed by educators were collected by the researcher before the start of the interview. Educators from Pécs, Zagreb and Masaryk universities were informed that the interview will be recorded and that the recordings would be deleted after transcription. Seven interviews were held in the Hungarian language, then translated into English, while the rest were held in English, and the recordings were transcribed verbatim. The interview recordings in Hungarian language were stored on the University of Pécs server, while those in English were kept on the Karolinska Institutet server. Both sets of recordings were retained for a few weeks and then permanently deleted after the data was transcribed. The interview guide was developed to answer the following research questions:

- What pedagogical knowledge and teaching practices are employed by educators who are teaching anatomy courses?
- What gaps do educators perceive at the institutional, departmental, and individual levels in relation to anatomy teaching?

This interview guide contained closed and open-ended questions covering the following areas: (i) socio-demographic characteristics and questions about teaching, for example gender, university degree, teaching experience in years and formal pedagogical training; (ii) teaching practices in relation to student-centred learning; (iii) knowledge on pedagogical frameworks such as CA and concepts such as ILOs; and (iv) potential gaps at the institutional, departmental and individual levels with regards to anatomy teaching. During data collection we used both close and open-ended questions to assure the appropriate of the data. Our study can be replicated, and it can be applicable to similar context.

### Data analysis

The transcribed data were kept in word document and stored in a file. Coding took place after cleaning the transcribed data from any words with no meaning and all names that could help to identify participants (e.g. name of a country). After coding in spreadsheet in a computer was completed, data were subjected to thematic analysis [18], which facilitated the identification, analysis, interpretation, and reporting of patterns or themes throughout the entire data set. This method provided a comprehensive description of the investigated phenomena and allowed for the interpretation of its various aspects. A theme, which encapsulates an essential aspect of the data in line with the overall research objective, indicates a patterned response or meaning within the data set. Data collected were kept safely and processed ensuring protection against unauthorised access.

The analysis was conducted by following six steps [19, 20]: Step (1) The interviews (data items) were transcribed word for word by the researcher (AE), then read and re-read multiple times so that the investigators could familiarise themselves with the data set. Step (2) Significant features across the data set were coded by two researchers separately, and data associated with each code were compared. A list of different codes was created from the entire data set, guided by the study aims. Step (3) At this stage, codes were categorised into potential broader subthemes/themes, and related data were compiled within each potential subtheme/theme. Step (4) All coded data extracts were reviewed again, and their structure under each subtheme/theme was evaluated. Consistent patterns within subthemes/themes were examined, and a thematic map of the analysis was created. Step (5) Data extracts that formed subthemes and themes were revisited, organised, and refined. The core of each subtheme/theme was identified and explained in the results section. Step (6) A detailed analysis was written for each subtheme/theme in relation to the research aim and within the context of the other themes (see results and discussion section). In this study, we have applied rigorous data collection methods as we used interviews and the thematic analysis that are well documented. In this study we achieved Triangulation by involving educators from several medical institutions in different countries, which helps mitigate the influence of local factors unique to a single institution.

**Fig. 2:**
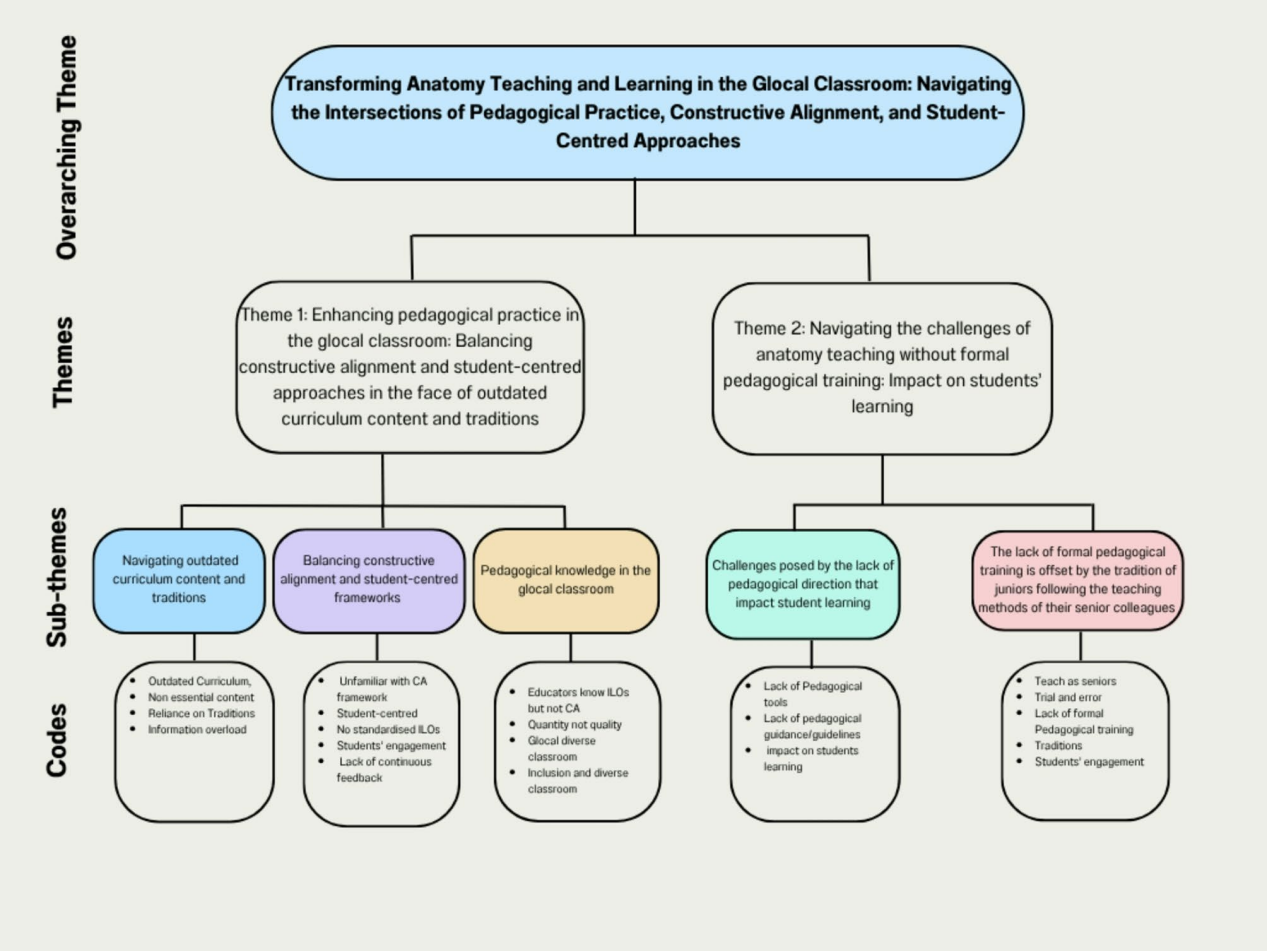
Overview of thematic analysis-modified in Canva

**Fig. 3:**
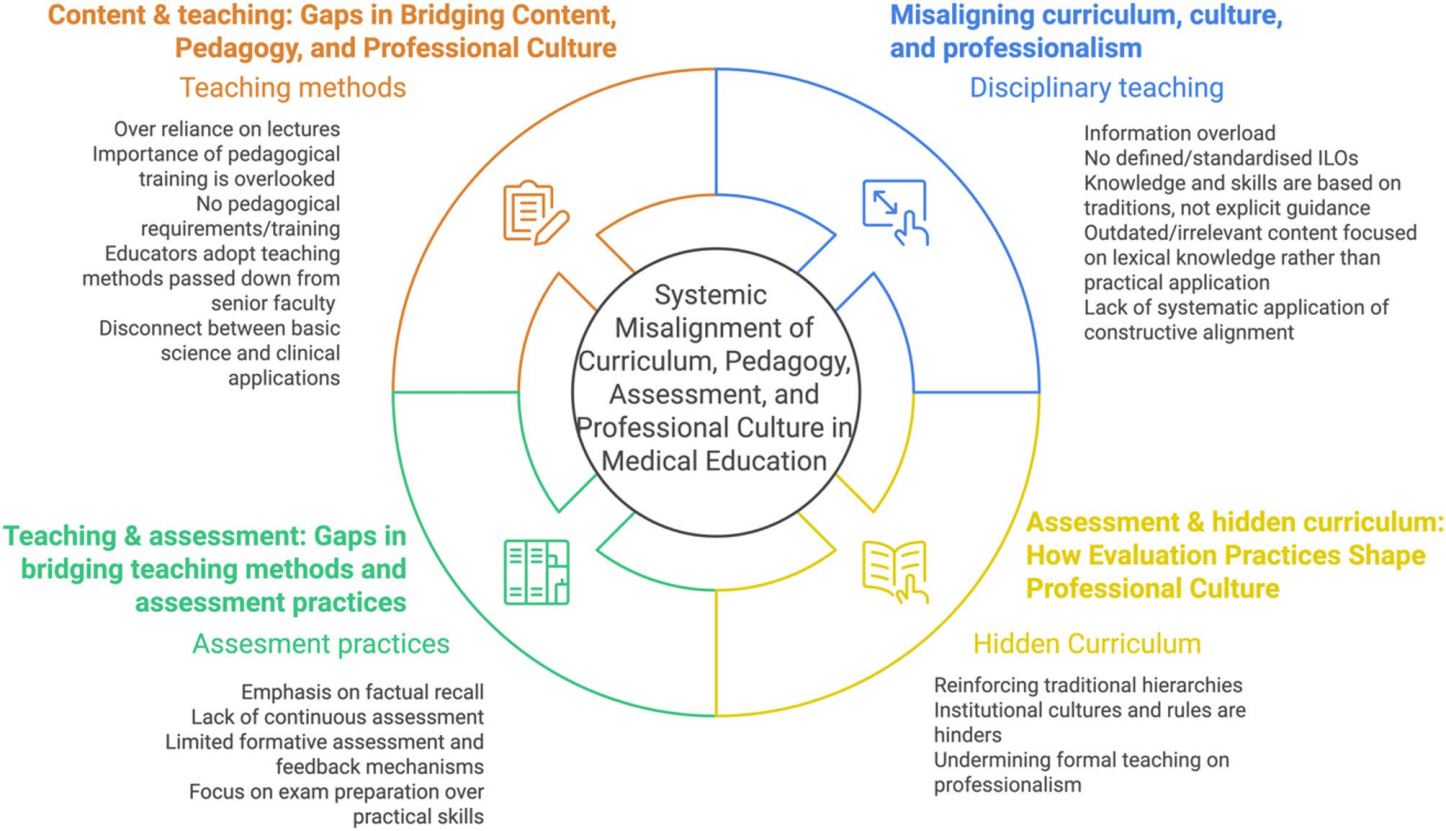
Perceived challenges and current problems of anatomy medical education-Figure created in Napkin

**Fig. 4:**
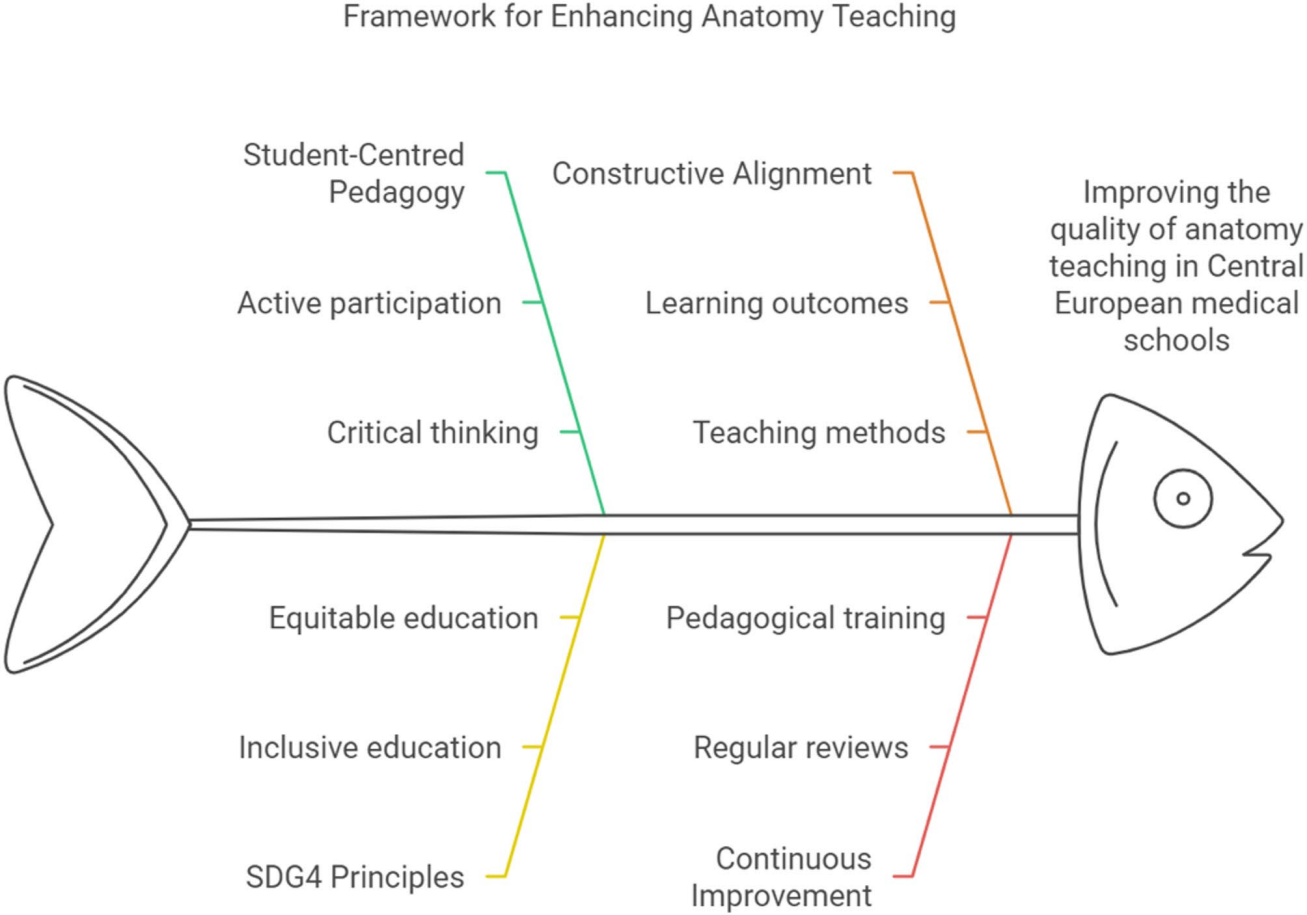
This is a fishbone graph representing a framework for enhancing anatomy teaching in central European medical schools-Figure created in Napkin

Figures 1, 3, and 4 feature images generated using Napkin, an AI-based image creation tool. These visuals were based on textual descriptions written by the authors and then adapted to illustrate main concepts discussed in the article. Figure 2 was modified using Canva, a graphic design platform, particularly to adjust the caption of the diagram. The authors accurately adapted the visualisations and handled the represented data critically. All images were carefully reviewed for accuracy and appropriateness with the research goals prior to publication. The use of Napkin and Canva complies with ethical guidelines, and all visuals are original creations free from copyright restrictions.

## Results and discussion

One overarching theme “Transforming Anatomy Teaching and Learning in the Glocal Classroom: Navigating the Intersections of Pedagogical Practice, Constructive Alignment, and Student-Centred Approaches” was identified from the data. Through thematic analysis, we identified one umbrella theme, two main themes, and five subthemes (three under theme 1 and two under theme 2), as illustrated in Fig. 2.

### Theme 1 enhancing pedagogical practice in the glocal classroom: balancing constructive alignment and student-centred approaches in the face of outdated curriculum content and traditions

#### Subtheme 1 navigating outdated curriculum content and traditions

Almost all respondents acknowledged a defined curriculum provided by their HEIs to outline the content to be covered in each class. However, several respondents identified certain aspects of the anatomy curriculum as outdated, irrelevant, or overly focused on lexical knowledge rather than practical application: *“It would be nice if we could filter out the information and the curricula that are completely outdated and have no use for medical students. So*,* the problem is that a lot of people still have in their minds that quantity is more important than quality*,* and that quantity is more important because they will forget about it anyway* [*…*] *The fact that we can’t get rid of traditions*,* traditions that are really quite outdated*,* that’s probably the biggest negative*,* and for me the lack of accountability”* (ED 3).

Respondents raised important concerns about the current state of medical education, particularly regarding an over-reliance on tradition. There appears to be a tendency to adhere to long-established practices and the legacy of historically influential faculty, even when these may no longer align with current international standards. Additionally, the curriculum is viewed as overly broad, potentially incorporating non-essential content that can overwhelm students. Consequently, there is a growing call among respondents to modernize the curriculum, emphasizing knowledge that is more relevant and essential for today’s medical students. These concerns reflect themes in recent academic literature, which discusses the challenges of information overload in medical education [21] and the need to address gaps in knowledge through reforms to traditional teaching methods [22].

#### Subtheme 2: balancing constructive alignment and student-centred frameworks

Nearly 70% of the educators were unfamiliar with CA and its underlying framework and expressed doubts about the specifics of this method. However, after being introduced to the definition of CA, most respondents concurred that this approach would be applicable to their teaching methods. One educator believed that CA assists in maintaining student engagement and motivation by clearly conveying the objectives and benefits of the learning activities. Nevertheless, the educator recognised the need for improvement in the systematic application of CA, especially in terms of providing effective feedback to students. Within CA, educators have some flexibility to adapt their lesson delivery and to improvise to a certain extent, depending on circumstances. While the structured curriculum and partial focus on student-centred learning align somewhat with the principles of CA, the lack of continuous assessment and feedback mechanisms represents a significant area where current practices diverge from the CA framework.

In relation to teaching objectives, CA supports the formulation of performance criteria that clearly define what students should be able to accomplish. For students, CA guides the design of exams to ensure they accurately assess the intended learning outcomes, thereby ensuring validity [17].

While most respondents were familiar with the term ILOs, they reported that no clear, standardised set of ILOs or minimum requirements were defined for their anatomy curricula. Instead, the expected knowledge and skills are shaped more by tradition and individual educator preferences than by a centralised, objective framework. The absence of continuous, weighted assessments contributing to the final grade is seen as a major gap, as it reduces student accountability and motivation [23]. One educator explained: *“It’s very important because we have a high failure rate in this subject*,* which can indicate various issues. One key issue is that students aren’t receiving quality feedback about their progress. This means a lot of time passes while they are off track*,* and it’s often not until exam time that we realize they’ve been struggling. By then*,* they have little time to catch up*,* which affects their chances of passing*,* let al.one achieving a good score. Implementing constructive alignment could help improve failure rates. It would be beneficial if it were applied systemically”* (ED 4).

Most respondents reported that the ILOs are often not documented nor are they formally written. Some respondents noted that ILOs are typically determined by senior professors during collaborative group discussions, where these senior faculty members decide what should be included in the ILOs, and the final approval is always required from the head of the department.: *“The intended learning outcomes are often written by the head of the unit or department but mainly kept for administrative purposes only*,* we revise them every three years”* (ED 9). Another respondent mentioned: “*In teams (teams: the working group that includes several anatomy teachers) at department*,* each team will be responsible to write ILOs*” (ED 1). When respondents were asked: do you revise ILOs and if so, who revises them? One respondent answered: *“If it will be revised it will be the leader”* (ED 12), while another said: “*It’s not written to be revised”. A respondent added: “Very rarely*,* it’s a tried and tested method*,* the anatomy doesn’t change much*,* so very rarely there are 1–2 changes at most*,* but not typical*” (ED 7). This indicates that different universities have different routines and traditions.

#### Subtheme 3 pedagogical knowledge in the glocal classroom

To explore the pedagogical knowledge of educators, a series of introductory questions were presented asking them about their familiarity with such pedagogical principles as CA, ILOs, and student-centred learning. They were presented with the following definition:

“Pedagogical knowledge means the knowledge of teachers and includes all the required cognitive knowledge for creating effective teaching and learning environments. Most research studies use the distinction between declarative (‘knowing that’) and procedural knowledge (‘knowing how’) from cognitive psychology as a theoretical basis. This approach is relevant as it focuses on understanding how knowledge is related to behaviour, or in other words, the quality of teaching performance” [24].

Educators in this study predominantly lacked comprehensive pedagogical knowledge, failing to recognize the critical distinction between ‘what to teach’ and ‘how to teach’. This latter aspect is essential for cultivating effective teaching and learning environments, particularly in enhancing the quality of anatomy education. Effective teaching transcends simple information transfer; it entails nurturing a profound understanding and enthusiasm for the subject matter. By doing so, educators can guide students to become active learners capable of applying their knowledge to real-world scenarios [24]. While this endeavour is undoubtedly challenging, implementing appropriate strategies and maintaining a dedication to continuous learning and improvement can yield immensely rewarding results [25].

This is particularly relevant in today’s multiculturally and linguistically diverse learning environments. All respondents confirmed that their student population is diverse, and that their anatomy education programme includes both local/national and international students, especially on the English-taught programmes. Some educators expressed concern over the lack of focus on inclusion and diversity in their institutions, stating: *“The teaching here is about quantity*,* not quality*,* no consideration for diversity or inclusion”* (ED 13). This quote underscores the reality that these HEIs are not in alignment with global initiatives such as SDG4.7, which emphasises the importance of being culturally and linguistically responsive in teaching. Such responsiveness is crucial for ensuring equitable and inclusive education that caters to the diverse needs of students from various backgrounds, ultimately enhancing the overall learning experience and outcomes in anatomy education. The concerns raised by these educators mirror the findings of the LEANbody needs analysis survey, which revealed that over 70% of anatomists (49/69) from a number of Central European universities have limited knowledge of international quality standards in medical education [11]. This observation aligns with research highlighting the deeply ingrained cultures within academic disciplines, which can lead to various challenges, including demographic underrepresentation, difficulties in addressing differences, and the presence of an implicit curriculum in anatomy education [26]. To address these issues, it is crucial for educators to develop glocal competences. This concept combines global awareness with local understanding, referring to an educator’s ability to interact effectively with students from diverse backgrounds while considering both global and local contexts. Glocal competences involve the ability to bridge global perspectives with local realities, ensuring that educational practices are both internationally relevant and locally appropriate. Educators with glocal competence can create inclusive learning environments that value diversity, promote cross-cultural understanding, and prepare students for success in an interconnected world [12].

### Theme 2 Navigating the challenges of anatomy teaching without formal pedagogical training: Impact on students’ learning

#### Subtheme 1 Challenges posed by the lack of pedagogical direction that impact students’ learning

Several respondents mentioned the lack of a clear, professionally grounded pedagogical direction or a set of guidelines as a significant challenge. They stated that many colleagues were perceived to be going in different directions, which makes it difficult for educators to implement evidence-based pedagogical approaches. The educators expressed a desire for more tools and training in effective pedagogical techniques: *“It would be nice if I had more tools*,* like a pedagogical direction that is professionally grounded. The question now is not whether the direction is well-established*,* but how well-known these professional guidelines are*,* and my experience is that they are not known among colleagues. Many colleagues are going in a different direction*,* which is perceived by the students*,* and so they take the attitude that why am I hanging out*,* why am I doing it differently from other colleagues. It would help a lot if there was a unified professional direction to go towards the best quality”* (ED 4). This subtheme shows clearly the essential need for pedagogical tools and comprehensive educator training develop educators who can positively impact student learning, foster a love of learning, and contribute to the overall success of the education system [27].

#### Subtheme 2 The lack of formal pedagogical training is offset by the tradition of juniors following the teaching methods of their senior colleagues

When educators shared their experience of when they first started teaching, they stated that they had to learn teaching skills and techniques through trial-and-error, without the benefit of a pedagogical foundation. Most of them had to rely on learning from senior colleagues and their own intuition. As the characteristics, qualities and attitudes of today’s students have changed, our respondents believe that more formal pedagogical training is needed to effectively engage and teach students. Many respondents reported being assigned teaching responsibilities without sufficient preparation. Instead of formal training, they relied on observing and learning from their more experienced colleagues’ teaching methods. One participant explained: *“Never any formal training. How to teach anatomy specifically or how to teach anatomy to medical students and how to teach anatomy to dental students and how to teach to anatomy to some other group of students. So this is something that we learn on the fly as basically you get thrown in and do your best regarding that. Of course*,* the Department of the Anatomy has a lot of people who are well versed in these things. So and we are like a tight knit group*,* so everyone basically is willing to help in these aspects. But there is like not formal training where someone takes. Yeah*,* you like*,* when you come at the department and then you are like studying a year under someone to how to teach.*” (ED 1). This subtheme highlights that the educators recognise the need for formal pedagogical training and guidance to improve their teaching practices and effectively address the diverse learning needs of students [27] in anatomy education, which is currently lacking in the discipline’s established tradition [28].

Despite the extensive teaching experience of the educators in this study, most of them lacked formal pedagogical training. Only 2 educators had received pedagogical training, one abroad and the other through a one-week online teaching course during the COVID pandemic. The absence of pedagogical preparation likely contributed to the challenging experiences faced by these educators. “*We have an educational tradition that doesn’t require pedagogical knowledge basically. It is a rather strict tradition*,* which means that as young teachers we have done things based on the experience of older teachers. At several points there were checks*,* we sat a lot of exams when we were young*,* where we saw the main pedagogical skills*,* the pedagogical skills used in exams*,* which were not specifically formulated*,* but still very different people at the institute level worked on similar principles and so you actually inherit this kind of abstraction of what are the principles that we teach on. That’s what we call a tradition*.” (ED 5). Consistent with findings from a recent study in Hungary [29], educators reported that there are no formal requirements for pedagogical training or knowledge at these institutions. Instead, new educators are expected to learn and adopt teaching methods and principles from senior faculty members. These institutions maintain a strong, established educational tradition that heavily influences their teaching approaches, placing little emphasis on external or standardized pedagogical frameworks [30]. As a result, teaching styles and strategies vary widely. One educator described their approach: *“I help them*,* but I’m also strict. I don’t lecture; I engage them in the lesson. Some students dislike that I call them by name in class. My lessons are interactive*,* with constant questioning. I try to demonstrate and come up with new ways to improve myself”* (ED 2).

In this way, quality medical education faces significant challenges due to misalignments between formal curricula, teaching methods, assessment practices, and the hidden curriculum that shape professional culture. These systemic issue impacts the quality and effectiveness of medical training, potentially compromising the development of well-rounded, competent healthcare professionals, as shown in Fig. 3 below.

The disconnect between curriculum content and teaching methods is a primary concern in medical education. Traditional lecture-based approaches remain prevalent, despite evidence supporting more interactive and engaging pedagogical techniques [31]. Many medical educators lack formal training in teaching methodologies, often relying on methods passed down from senior faculty without critical evaluation of their effectiveness. Furthermore, there’s a noticeable gap between basic science instruction and clinical application. This disconnect can leave students struggling to apply theoretical knowledge in practical settings, hindering their ability to develop crucial clinical reasoning skills.

The hidden curriculum, comprising unwritten rules and cultural norms, significantly influences students’ professional development. This informal learning environment often reinforces traditional hierarchies and institutional cultures that may contradict the formal teachings on professionalism. For instance, students might observe behaviours from senior clinicians that conflict with the ethical standards taught in classrooms, leading to confusion and potential erosion of professional values [32].

By addressing these systemic misalignments, medical education can evolve to better prepare future healthcare professionals for the complex, dynamic healthcare environment they will navigate. This holistic approach ensures that formal curricula, teaching methods, assessment practices, and professional culture work in harmony to produce competent, ethical, and adaptable medical practitioners.

### Recommendations for central european medical schools

The LEANbody project investigated strategies to enhance the quality of anatomy teaching in Central European medical schools. To address this challenge, we propose an integrated approach that combines four key educational principles: student-centred learning, internationalisation of education, Sustainable Development Goal 4.7 (SDG4.7), and constructive alignment (CA).

### Conclusions and future directions

Student-centred learning places learners at the heart of the educational process, encouraging active participation and critical thinking. This approach aligns well with SDG4.7, which focuses on promoting inclusive, equitable, and quality education for all, while emphasizing global citizenship and sustainable development. Internationalisation of education broadens students’ perspectives and prepares them for a globalised healthcare environment. Constructive alignment ensures that learning outcomes, teaching methods, and assessments are coherently linked to enhance educational quality. The intersection of these concepts is expected to create a framework for improving anatomy education as shown in Fig. 4 below.

By integrating these elements into the curriculum, we can create a learning environment that not only enhances the quality of anatomy education but also empowers students to become knowledgeable, skilled, and engaged global citizens. This approach requires regular pedagogical training for teachers to ensure effective implementation and adaptation to evolving educational needs.

To implement this integrated approach, medical schools should:

1. Provide comprehensive training for educators on student-centred active learning techniques, internationalisation strategies, and the principles of SDG4.7.
2. Develop clear guidelines for incorporating these principles into anatomy course design and delivery.
3. Establish mechanisms for ongoing evaluation and improvement of teaching practices.
4. Foster collaboration between anatomy departments and international partners to enhance global perspectives in the curriculum.

This integrated approach could potentially generate a more responsive, inclusive, and effective educational experience that prepares students for the complex challenges of modern healthcare and sustainable development in a globalised world. A near-future follow-up project incorporating regular formal educators training and subsequent evaluation shall be considered. This shall be aligned with implementing the suggested frameworks for improving anatomy teaching in medical schools across Central Europe.

### Limitations of the study

This study had a limited number of educators due to the inclusion criteria, which restricted participation to members of the LEANbody project. This limited number of participants may not fully capture diversity of perspectives teaching practices, and institutional contexts present within and across central European universities. As a result, the findings may have limited generalizability and may not reflect the broader experiences or challenges faced by anatomy educators outside the project or at other institutions. For future research, it would be advantageous to involve more institutions and anatomy educators to broaden the participant pool. These results may not be generalizable. Researchers with different reflexivity than those conducting this study might uncover aspects not identified in this research.

## Supplementary Information

Supplementary Material 1.

Supplementary Material 2.

## Data Availability

The datasets used and/or analysed during the current study are available from the corresponding author on reasonable request.

## Abbreviations

CA: Constructive alignment is an educational principle that ensures coherence between intended learning outcomes, teaching activities, and assessment methods to optimize student learning and achievement.
ILOs: Intended learning outcomes are clear, specific statements that describe what students should know, understand, and be able to demonstrate upon successful completion of a course or program
SDGs: Sustainable development goals are a set of 17 interconnected global objectives established by the United Nations in 2015 to address pressing environmental, social, and economic challenges, aiming to create a more sustainable, equitable, and prosperous world for all by 2030.
SDG4: Sustainable Development Goal 4 aims to ensure inclusive and equitable quality education and promote lifelong learning opportunities for all.
SDG4.7: Sustainable Development Goal 4 target 7 aims to ensure that by 2030, all learners acquire the knowledge and skills needed to promote sustainable development, including education for sustainable development, sustainable lifestyles, human rights, gender equality, promotion of a culture of peace and non-violence, global citizenship, and appreciation of cultural diversity.
TLAs: Teaching and learning activities are structured educational experiences designed by instructors to help students achieve specific learning outcomes through active engagement with course content, concepts, and skills.
