# Supplementary material for: Anatomy education at central Europe medical schools: a qualitative analysis of educators’ pedagogical knowledge, methods, practices, and challenges
Source: BMC Med Educ. 2025 Aug 18;25:1173. doi: 10.1186/s12909-025-07722-6 (PMC12359980; doi:10.1186/s12909-025-07722-6)
Supplement: Supplementary file 1 — Supplementary Material 1. [file 12909_2025_7722_MOESM1_ESM.pdf]

Consent Form for Explore pedagogical knowledge of teachers teaching anatomy courses in Zagreb, Pécs and Masaryk Universities

Please tick the appropriate boxes	**Yes**	**No**
**Taking part in the study**		
I have read and understood the study information dated [DD/MM/YYYY], or it has been read to me. I have been able to ask questions about the study and my questions have been answered to my satisfaction.	○	○
I consent voluntarily to be a participant in this study and understand that I can refuse to answer questions and I can withdraw from the study at any time, without having to give a reason.	○	○
I understand that taking part in the study involves an audio-recorded interview and that these interviews will be transcribed as text, and the recording will be destroyed after the research has been completed.	○	○
**Use of the information in the study**		
I understand that information I provide will be used for a qualitative research	○	○
I understand that personal information collected about me that can identify me, such as [e.g. age, study background], will not be shared beyond the study team, and that data will be coded and anonymised.	○	○
I agree that my information can be quoted anonymously in research outputs	○	○

Signatures

______________________ ______________________ ________

Name of participant [printed] Signature Date

Study contact details for further information: [Dr. Amani Eltayb, Karolinska Institutet; amani.eltayb@ki.se]
