# Supplementary material for: Anatomy education at central Europe medical schools: a qualitative analysis of educators’ pedagogical knowledge, methods, practices, and challenges
Source: BMC Med Educ. 2025 Aug 18;25:1173. doi: 10.1186/s12909-025-07722-6 (PMC12359980; doi:10.1186/s12909-025-07722-6)
Supplement: Supplementary file 2 — Supplementary Material 2. [file 12909_2025_7722_MOESM2_ESM.pdf]

Interview guide

-Anonymous individual interview (it's necessary that the interviewer will assure to the interviewee that it will be anonymous)

The interviewer explains aims of the study: To understand educators' perceptions of pedagogical knowledge and concepts/frameworks, such as constructive alignment (CA), Intended Learning Outcomes (ILOs), in relation to student-centred pedagogy and their anatomy teaching practices. The study also investigates perceived gaps at the institutional, departmental, and individual levels concerning anatomy teaching and the pedagogical practices that should be promoted. This study raises questions considering teaching in today's multicultural and multilingual classrooms as: Are anatomy educators adequately prepared to provide high quality teaching and learning that align with global educational standards and sustainability agendas? Are these educators formally trained to equip graduates with the necessary knowledge, skills, and attitudes for a globalised world? Are they aware of pedagogical approaches and principles such as student-centred learning, constructive alignment (CA), and ILOs among others? Do they apply these pedagogical principles to their practices? What are the potential gaps as perceived by educators at the institutional, departmental, and individual levels with regards to anatomy teaching?

To answer these questions, we will conduct this study to:

- What pedagogical knowledge and teaching practices are employed by educators who are teaching anatomy courses?
- What gaps do educators perceive at the institutional, departmental, and individual levels in relation to anatomy teaching?

Interviewer explains (Pedagogical knowledge means the knowledge of teachers includes all the required cognitive knowledge for creating effective teaching and learning environments. Most research studies use the distinction between declarative ('knowing that') and procedural knowledge ('knowing how') from cognitive psychology as a theoretical basis. This approach

is relevant as it focuses on understanding how knowledge is related to behaviour, or in other words, the quality of teaching performance.) Ref: https://www.oecd.org/education/ceri/Background_document_to_Symposium_ITEL-FINAL.pdf

- Informed consent: Your participation is voluntary, confidential, and that you could withdraw at any time. Your responses will be coded, analysed, and used for publication purposes. No individual educators will be identifiable in the data. The recordings of the interviews will be deleted permanently immediately after transcription of the data. The data is accessible only to the study authors on a password-protected Karolinska Institutet university server and will be stored for ten years before deletion, as required by the European Commission, which funded the LEANbody project.

Written Consent: ask participant to sign it (https://kise-my.sharepoint.com/personal/amani_eltayb_ki_se/Documents/Skrivbordet/Leanbody%20manus/Submission/Consent%20Form%20for%20Explore%20pedagogical%20knowledge%20of%20teachers%20teaching%20anatomy%20courses%20in%20Brno%20University%20-%20Copy.pdf)

Its vital that the interviewer try to probe as much as he/she/they can

General/warm-up

Tell us about yourself, age, family, … Ice breaking question? teaching qualifications?

Questions on participants' teaching

Describe your teaching session Let them talk then start according to the interview guide

1. How do you prepare for your teaching?
2. What are the challenges you face in your teaching?
3. How do you deal with these challenges?
4. Describe a teaching session when you have experienced difficulties? How did you deal with these difficulties?

Knowledge on constructive alignment (CA)

5. Have you ever heard of constructive alignment in teaching?

YES ☐ NO ☐ If the answer was YES, ask from whom?

6. Could you explain what is constructive alignment? Constructive alignment (CA) is **an outcomes-based approach to teaching in which the learning outcomes that students are intended to achieve are defined before teaching takes place**.

7. Describe How do you apply it in your teaching?

8. If She/he/they answer NO then the interviewer explains what it is let them reflect and express their views in regards to their teaching?

9. Do you know what is intended learning outcome (ILOs)? YES ☐ NO ☐

If She/HE/They answer No explain and let them reflect?

If She/HE/They answer YES ask from where you hear/know about?

Explain what it is?

10. Describe how the process with regards to ILOs is at their institution? Whether they work with programme/course ILOs and how

1. Probing questions if needed Do you have access to the programme Learning outcomes? YES ☐ NO ☐

2. Do you use ILOs when you prepare for your teaching sessions? YES ☐ NO ☐

3. Do you write the ILOs of your teaching sessions yourself? YES ☐ NO ☐

    1. If the answer was NO then ask who and why?

4. If you write the ILOs yourself, do you revise them?

YES ☐ NO ☐ and if the answer was YES why do you revise them and how often?

11. Describe what you do and how do you to make students engaged or active during your teaching sessions? You can provide examples?

12. Describe how do you assess your students? How frequently?

13. Describe how do you measure their learning or understanding during your teaching sessions?

14. Tell us about your thoughts with the level of teaching and learning at your institution/department? In other words, probe if they have any motivation for change? Then ask

15. Tell us What would you like to do differently and why
